# Pharmacokinetic Characterization of Labetalol in Pregnancy (The CLIP Study): A Prospective Observational Longitudinal Pharmacokinetic/Pharmacodynamic Cohort Study During Pregnancy and Postpartum

**DOI:** 10.3390/jcm14082793

**Published:** 2025-04-18

**Authors:** Surya Bhamidipaty-Pelosi, Suhaas Muralidharan, Brittany C. Yeley, David M. Haas, Sara K. Quinney

**Affiliations:** 1 Division of Clinical Pharmacology, Department of Medicine, Indiana University School of Medicine, Indianapolis, IN 46202, USAdahaas@iu.edu (D.M.H.); 2 Department of Pharmacy Practice, Purdue University College of Pharmacy, West Lafayette, IN 47907, USA; 3 Department of Obstetrics and Gynecology, Indiana University School of Medicine, Indianapolis, IN 46202, USA

**Keywords:** hypertensive disorders of pregnancy, labetalol, chiral LC-MS/MS, population PK/PD model, protocol

## Abstract

**Background/Objectives:** Hypertensive disorders of pregnancy are a leading cause of pregnancy-related deaths in the United States, accounting for 7% of maternal mortality. Labetalol and nifedipine are the first-line drugs for the management of hypertension in pregnancy, but there are little data guiding the choice of one drug over the other. The current pilot longitudinal study aims to characterize the pharmacokinetics (PK) and pharmacodynamics (PD) of labetalol stereoisomers throughout pregnancy and postpartum. **Methods:** This is a single-center clinical study recruiting up to 40 pregnant individuals ≥ 18 years of age at the time of enrollment, taking labetalol as per the standard of care. The exclusion criteria include any pathophysiology impacting the PK of labetalol, e.g., liver failure. Maternal plasma, urine, amniotic fluid, cord blood, and breast milk will be collected, and labetalol stereoisomers will be measured using a validated LC-MS/MS assay. Heart rate and blood pressure will be measured as the PD endpoints. These may be assessed throughout a participant’s dosing interval at scheduled PK study visits, which will occur every 6–10 weeks during pregnancy, at delivery, during the 1st week postpartum, and up to 20 weeks postpartum. The primary aim is to characterize the PK and PD of labetalol during pregnancy and in the postpartum period. The secondary aim is to determine the extent of breast milk excretion of and infant exposure to labetalol from breast milk. The data will be analyzed using population PK modeling to evaluate the PK/PD relationship and ultimately develop trimester-specific dosing recommendations. **Results/Conclusions:** To our knowledge, this is the first study aiming to characterize the PK of labetalol stereoisomers across pregnancy and postpartum, utilizing individual stereoisomer data to evaluate the PK/PD relationship, and collecting postpartum samples, including breast milk, to model infant exposure to labetalol through breast milk. This study will provide important PK/PD data and knowledge which will be combined with large multi-center clinical trial data to develop trimester-specific dosing regimens for anti-hypertensive agents.

## 1. Introduction

Hypertensive disorders of pregnancy (HDPs) are among the most common medical comorbidities in pregnancy, and include chronic hypertension, gestational hypertension, preeclampsia/eclampsia, and chronic hypertension with superimposed preeclampsia/eclampsia [1,2]. Globally, HDPs affect roughly 10% (or 18 million) of all pregnancies annually [1], and are a leading cause of pregnancy-related deaths. In the United States, HDPs account for 7% of maternal mortality cases, with roughly 70% of these deaths occurring postpartum [3,4]. Pharmacologic treatment is the cornerstone of HDPs management, with the early and adequate control of blood pressure (BP) associated with improved maternal and neonatal outcomes [5,6].

Labetalol and nifedipine are the first-line drugs for the management of hypertension in pregnancy [5], but there is limited evidence on the optimal dosing during pregnancy. Labetalol has been shown to be safe and effective for the treatment of hypertension in pregnancy [5,6,7,8]. However, the absence of a unified global dosing recommendation contributes to discrepancies in treatment approaches. This is further complicated by the limited data available to advise on the optimal dosing for pregnancies with comorbidities, e.g., obesity and renal disease. Further, different organizations provide varying recommendations for the maximum daily doses, e.g., the World Health Organization (WHO): 2500 mg/day [9]; the American College of Obstetricians and Gynecologists (ACOG) and Queensland Health Clinical (QCH): 2400 mg/day [10,11]; and Chinese guidelines: 600 mg/day [12]. Clinically, patients and healthcare providers alike would benefit from evidence-based trimester-specific dosing recommendations. Labetalol acts by selectively blocking alpha-1 adrenergic and nonselectively blocking beta-adrenergic receptors. This action leads to a reduction in both the heart rate (HR) and BP [8,13,14]. Labetalol is a chiral drug with two diastereomeric pairs of racemates, and each stereoisomer has distinct activity [15,16]: (R,R)-labetalol is an active nonselective β-adrenoreceptor antagonist, while (S,R)-labetalol is largely responsible for α1-antagonist activity [17]. (R,S)- and (S,S)-labetalol have minimal activity [15,16].

The pharmacokinetics/pharmacodynamics (PK/PD) of racemic labetalol in non-pregnant individuals are well described [7,18]. Labetalol has a relatively short half-life (5–6 h) and a complex metabolism that is not well characterized. Following oral administration, labetalol is readily absorbed, achieving peak plasma concentrations within 60–90 min. Labetalol is an intermediate hepatic extraction ratio drug and undergoes extensive first-pass metabolism, resulting in an average bioavailability of 20–35%. It is moderately protein-bound (50–60%), with an elimination half-life of approximately 6–8 h [7,18]. Very little unchanged labetalol is excreted in urine. Of note, labetalol is known to exhibit a significant inter-patient individual variability that can range from 11–86% [7,18,19,20]; however, the reason for this has not been explored.

Improving our understanding of the stereoisomer-specific PK and PD changes in pregnancy is important, given that the early and adequate control of BP is associated with improved maternal/fetal outcomes. Both the β- and α1-blocking properties of labetalol are beneficial for treating hypertension in pregnancy. The PK/PD properties of most beta-blockers have been described and summarized by Mehvar et al. [21]. Stereoselectivity contributes to wide variations in the PK, which may also be reflected in the observed variable PD responses. The PK of intravenous and oral labetalol during pregnancy and postpartum have been evaluated, albeit, in a handful of studies which had small sample sizes, e.g., 7–9 pregnant patients/study [22,23,24,25,26]. Stereoselective metabolism and disposition have been observed in oral dosing but not in intravenous dosing, indicating that a higher labetalol first-pass metabolism and lower bioavailability during pregnancy following oral administration may be driven by the active (R,R)-labetalol stereoisomer [22]. Studies have demonstrated stereoselective PK following oral administration, such that the apparent oral clearance values for the active (R,R)-labetalol were higher than the less active (S,S)-labetalol [22]. However, these studies were limited by their lack of longitudinal data, e.g., during pregnancy and postpartum for the same individuals, and a lack of parallel non-pregnant female controls to serve as a direct comparison. Together, these data limitations result in qualitative comparisons relative to the historical literature derived from non-pregnant female and male subjects [23].

The physiologic changes that occur during pregnancy alter drug PK, and drug exposure during pregnancy can be drastically altered due to changes in maternal physiology [27]. Alterations in drug-metabolizing enzyme activity can lead to increased clearance (e.g., for CYP3A, CYP2D6, and UGT substrates) [27,28,29]. In fact, alterations in drug exposure during pregnancy often necessitate changes in drug dosing to maintain unbound drug concentrations within the therapeutic range [30,31,32,33]. Khatri et al. performed in vitro studies evaluating labetalol glucuronidation using human hepatocytes and demonstrated that (1) pregnancy-related hormones alter the function of UGT proteins in an isoform-specific manner in human hepatocytes, and (2) cause an increase in UGT1A1-mediated labetalol metabolism by inducing UGT1A1 protein concentrations [34]. Their work depicted a mechanistic rationale for the associated increases in labetalol clearance observed during pregnancy, which is consistent with other studies [27,29,34,35]. However, at present, analytical standards and reproducible methodology preclude the quantification of absolute concentrations of distinct labetalol glucuronides, and the fractional contribution of UGT1A1 and UGT2B7-mediated metabolism to labetalol clearance in vivo remains unknown [23].

The current study will characterize the PK and PD of labetalol stereoisomers antenatally and postpartum, as it is likely that the individual stereoisomers undergo stereoselective disposition. This clinical study’s design leverages a population PK (PopPK) analysis to evaluate PK changes antenatally and postpartum. The secondary aims are to determine the extent of breast milk excretion of and infant exposure to labetalol from breast milk. The exploratory aims are as follows: (1) to understand the sources of inter- and intra-individual variability in plasma and breast milk labetalol concentrations in this population, (2) to assess the relationship between the PK and PD (BP and HR) in pregnant and postpartum individuals taking labetalol, and (3) to determine the disposition of the labetalol in cord blood and amniotic fluid at delivery.

## 2. Study Aims

### 2.1. Primary Aims

The primary study aim is to characterize and quantify the PK of labetalol stereoisomer concentrations across pregnancy and postpartum using the validated methodology for chiral separation, LC-MS/MS, as described below.

### 2.2. Secondary Aims

The secondary aims are to determine the extent of breast milk excretion of and infant exposure to labetalol from breast milk.

### 2.3. Exploratory Aims

The exploratory aims are as follows: (1) to understand the sources of inter- and intra-individual variability in plasma and breast milk labetalol concentrations in this population, (2) to assess the relationship between the PK and PD (BP, HR) in pregnant and postpartum individuals taking labetalol, and (3) to determine the disposition of the labetalol in cord blood and amniotic fluid at delivery.

### 2.4. Demographic and Clinical Information

The members of this clinical study team will ascertain the demographic information available through electronic health records chart reviews and/or participant interviews. A full list of the variables to be assessed is included in Appendix A. The clinical information will be updated at each PK study visit to account for pregnancy-induced medical diagnoses and/or changes in medications.

## 3. Study Procedures

### 3.1. Population/Recruitment

**Figure 1:**
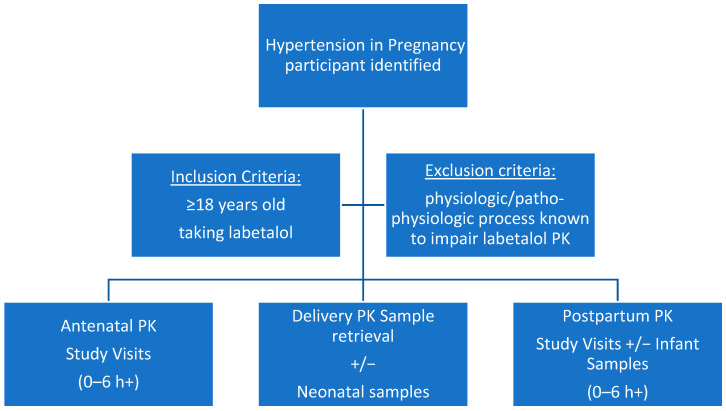
Inclusion criteria and study visit timing. The figure displays the inclusion and exclusion criteria for this study and the three timepoints for the PK study visits. Antenatal PK sampling is anticipated to occur during each trimester, with 6–10 weeks between visits. Postpartum sampling will be up to 20 weeks post-delivery.

Inclusion and exclusion criteria are presented in Figure 1. Pregnant women at any gestational age, older than 18 years of age, and taking labetalol as part of standard of care are eligible for recruitment. This study’s participants will be identified by Cerner, Epic, or treating clinician at Eskenazi Health or Indiana University Health Riley and University Hospitals. Recruitment commenced in January 2024 and will recruit up to 40 participants. Indiana University and Eskenazi Health Systems provide access to a diverse population of pregnant patients. Eligibility will be confirmed by this study’s team to rule out contraindications to participating in this study, e.g., hepatic impairment known to alter the PK of labetalol. Participants who meet all eligibility criteria will be provided with a copy of the IRB-approved informed consent to review. Optional neonatal enrollment will allow for collection of heel-stick blood sampling from neonates. All individuals who choose to enroll in this study will be asked to sign and date the informed consent document. All consenting participants will be provided with a photocopy of the signed consent form. This study was approved by the Indiana University IRB on 20 October 2023 (IRB#18956), and enrollment has begun.

### 3.2. Labetalol Dosing

Participants are only eligible for this study if they are prescribed and taking labetalol as part of standard of care for their hypertension. Labetalol is a commonly used first-line drug for chronic or acute hypertension treatment in pregnancy. The decision for a patient to be placed on labetalol is not influenced by this study’s investigators. This study’s team will only be alerted about a potential participant if they have already been prescribed labetalol. In our current population, roughly half of the patients with chronic hypertension are prescribed labetalol and the other half nifedipine. Treatment decisions are fully up to the care provider and patient in a shared decision-making care model. All potential participants will either be contacted by a study investigator or present themselves through self-referral to a member of this study’s team. Labetalol dosing may vary amongst participants, and participants will be asked to accurately record time and dose administered for 3 or more doses prior to the dose taken during their study visit. Details will be reviewed and confirmed prior to each clinical PK study visit. If a patient’s provider switches their medication to a drug other than labetalol during the pregnancy, the individual’s participation in future study visits will be halted, but the patient will not be withdrawn from this study, and any data collected to date will be analyzed. Additionally, if a participant is later changed back to labetalol, study activities may resume. Patients who are on multiple drugs for hypertension are eligible to be included in the study as long as one of the drugs is labetalol.

### 3.3. PK Clinical Study Visits

#### 3.3.1. Study Design

This open-label pilot observational longitudinal study will characterize the PK and PD of labetalol stereoisomers across pregnancy and in a postpartum setting. PK study visits will occur every 6–10 weeks during pregnancy (e.g., each trimester), during the inpatient postpartum stay, and up to 20 weeks post-partum, as long as the individual is taking labetalol. Delivery samples may be collected to assess maternal and fetal exposure to labetalol during parturition. PK study visits will be scheduled at the convenience of the enrolled participant and may occur at the Indiana University Clinical Research Center (CRC) and/or as a hospital inpatient.

#### 3.3.2. PK/PD Study Visit Rationale

**Figure 2:**
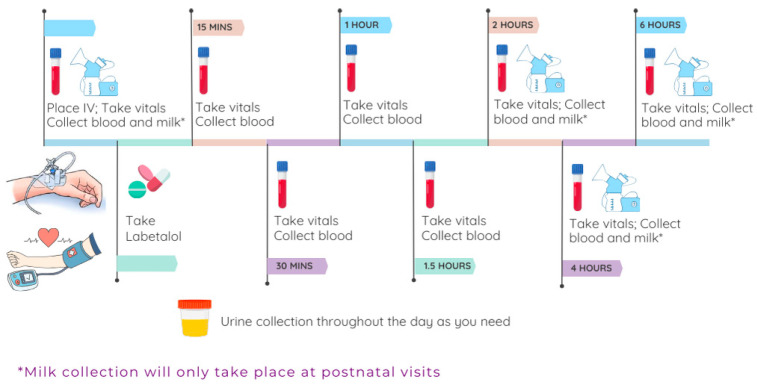
Clinical study visit flow. This figure displays the flow of activities during a clinical PK study visit, beginning with the recording of vital signs, blood draws, and other specimen collection (with breast milk collection only during the postpartum visit).

This PK/PD study is designed to assess labetalol plasma exposure through serial blood sampling with corresponding PD. To attain a comprehensive dataset, each clinical study visit will include serial blood sampling up to 6 h post dose. If the participant is a hospital inpatient, serial blood sampling and corresponding PD parameters (Figure 2) will be ascertained across the entire dosing interval.

#### 3.3.3. PK/PD Study Visit Sample Collection

PK study visits will be scheduled with respect to the participant’s availability and their labetalol dosing schedule. PK study visits can range between 4–6 h for CRC-scheduled study visits, and up to 24 h for PK study visits occurring for enrolled hospitalized inpatient participants. Blood draws will typically be across a single dosing interval. If a patient receives different doses throughout the day, and participant consent is attained, we may capture a full 24 h interval. Details regarding clinical study visits processes can be seen in Figure 2 and are outlined below.

Upon commencing a clinical study visit, participants will be asked to void their bladders and hold their labetalol medication until pre-dose vitals/blood samples are taken. An intravenous cannula will be placed by a trained nurse or phlebotomist, the participant will remain supine for 5 min, and baseline PD vital signs will be taken, which will include BP, HR, temperature, and respiratory rate. Of note, BP and HR will be measured by the same observer using a mercury sphygmomanometer. Participants will be asked to provide an extra dose (if available) so that we can assess variability in dosing between manufacturers, given the FDA’s allowance for 80–125% variability between compounds.

Participants may void as needed throughout their clinical study visit, and total urine volume will be collected and aliquoted for specimen analysis. Serial HR and BP and blood sampling into EDTA vials will occur during PK study visits at the following time points: pre dose, and then 15 min, 30 min, 1 h, 1.5 h, 2 h, 3 h, 4 h, and (optionally) 6 h post dose. Samples may be collected by venipuncture or through a study-specific intravenous catheter, depending on participant’s preference. If the participant’s clinical study visit occurs during hospitalization as part of medical standard of care, serial blood sampling and urine specimens will be collected for longer duration, up to 24 h.

Treatment notes and medications administered for all participants in clinical PK study visits will be ascertained prior to the start of each study visit. Delivery samples will include maternal plasma, venous umbilical cord blood, and amniotic fluid (in cases where delivery is by cesarean and membranes are unruptured at the time of delivery, and/or amniotomy as possible as per standard of care for enrolled participants electing for vaginal delivery). Among postpartum participants, breast milk and infant blood samples attained from capillary heel sticks may be collected to assess infant exposure to labetalol and/or metabolites through breastmilk.

#### 3.3.4. Pharmacogenomic Sampling

During the first PK clinical study visit, maternal blood will be collected at baseline for DNA extraction and genotyping/pharmacogenomic testing. Up to 3 mL of maternal blood will be drawn in an EDTA tube. This study includes optional participation of the infant, with opportunities to collect buccal swab for future pharmacogenomic evaluation if capillary heel-prick sampling or umbilical cord blood ascertained at delivery is insufficient for testing. Additional capillary heel-prick sampling (up to 1–2 mL) to assess infant exposure to labetalol through breast milk is optional.

## 4. PK Sample Analysis Plan

### 4.1. Biospecimen Collection, Processing, and Storage

All the maternal and neonatal PK samples will be collected at prespecified times (see Figure 2) and in vials labeled, respectively. The maternal samples will include urine, plasma, a blood sample for genotyping, breast milk, amniotic fluid, and cord blood. The neonatal samples will include plasma samples attained from heel-stick blood samples if provided, and/or a buccal swab for genotyping. All the samples will be protected from light (e.g., tubes wrapped in foil, an amber urine jug, and amber cryovials), and kept on ice until processed. All the samples, e.g., urine, plasma, amniotic fluid, and breast milk, will be frozen at −70 °C until batch analysis. If obtained, the neonatal buccal swabs will be stored at room temperature for future pharmacogenomic testing.

During the PK study visits, all the samples will remain on ice and be light-protected to prevent sample and labetalol degradation. Plasma sample processing will occur throughout the clinical study visits.

The total volume of maternal urine collected throughout the duration of a study visit will be noted. The urine will be mixed manually and stored in 500 µL aliquots in light-protected vials. All the blood sample processing will include centrifugation at 1800× *g*, 4 °C for 15 min; the supernatant plasma sample will be extracted and stored in 500 µL aliquots in light-protected vials. Amniotic fluid and breast milk samples will be collected and directly aliquoted in 500 µL light-protected vials.

### 4.2. Chiral Separation LC-MS/MS

The PK samples will be analyzed by the Indiana University Simon Comprehensive Cancer Center (IUSCCC) Clinical Pharmacology Analytical Core Laboratory (CPAC) using a validated LC-MS/MS chiral assay to quantify each stereoisomer. The method’s validation will follow FDA and ICH guidelines [36,37]. The CPAC has experience with chiral assays (e.g., bupropion) [38]. The assays will be validated against each sample matrix (plasma, breast milk, urine, amniotic fluid, etc.). This study will measure the individual stereoisomers of labetalol and depict the PK changes across pregnancy and postpartum.

## 5. PK Analytical Plan

### Population PK/PD Models

PopPK modeling techniques will be employed to characterize the disposition of the drug in the plasma, breast milk, and amniotic fluid. PopPK utilizes nonlinear mixed-effects modeling to determine the PK of each labetalol stereoisomer across pregnancy and postpartum. PopPK is a statistical method that allows for missing or sparse data, variability in the dose amount and dosing times, and can integrate data from both plasma and breast milk. PopPK analyses will be performed using NONMEM v.7.5 (ICON Development Solutions) [39] using PsN (version 5.5.0) and Xpose 4 (4.7.3) for model assessment and evaluation [40]. Proportional, additive, and combination error models will be assessed. Once a satisfactory structural model is determined, the covariates, including the maternal demographic and clinical data, gestational age at delivery, and time post-delivery, will be evaluated. The covariates will be evaluated based on their biological plausibility and retained in the model if the objective function value (OFV) decreases ≥ 3.84 (*χ*^2^-distribution, *p* < 0.05) on forward selection and increases > 10.83 (*p* < 0.001) on backward elimination. The model selection will be based on a comparison of the minimum OFV, evaluation of the population’s fixed- and random-effect parameter estimates, goodness-of-fit diagnostic plots, and visual predictive checks. A boot-strap analysis will be used to evaluate the stability and performance of the final model. Once the PK model is established, the PD measures (BP and HR) will be added to the model using PK/PD modeling approaches.

As this is a pilot and descriptive study, no formal sample size calculation has been performed. However, we aim to recruit 40 participants, as similar PK studies have typically enrolled between 12–30 individuals. A sample size of 40 should provide sufficient data to assess general trends in the PK over the gestational and postpartum periods, accounting for missing data and drop-outs. This study will also provide preliminary data to inform future studies for evaluating covariates (e.g., weight/body composition, pharmacogenomics).

## 6. Discussion

Optimizing the management of hypertension in pregnancy is of critical importance, due to its key role in maternal/neonatal morbidity and mortality. Currently, there is a paucity of clinically useful data on the dose optimization of anti-hypertensives during pregnancy and postpartum. There are few reported studies on PK during pregnancy and the placental transfer of labetalol-assessed absorption, distribution, metabolism, and excretion in pregnant women; however, these studies used different outcome measures for the PK, precluding data clinical translation. Furthermore, there are very few studies depicting labetalol PK/PD stereoisomeric disposition. In general, beta-blockers have demonstrated significant stereoselectivity in their pharmacologic effects, and stereoselective changes in the PK of these drugs may be associated with altered PD profiles. Thus, an understanding of the stereospecific PK and PD of labetalol may help clinicians interpret and predict differences among patients in their pharmacologic responses to these drugs. We present our protocol for the first clinical study evaluating the PK of labetalol stereoisomers throughout pregnancy and postpartum to depict a qualitative characterization of stereoisomer disposition. The results from the current study will form the basis for future trials, by (1) determining the dosing of labetalol for trimester-specific recommendations, and (2) providing biospecimens for future pharmacogenomic evaluation.

## Data Availability

There are no data available at present in support of the clinical study protocol proposed in this manuscript.

## Appendix A

**Table A1:** Data Collection from EHR or Patient Interview.

Maternal Demographics	
	Date of birth
	Race/ethnicity
	Smoking status
	Alcohol use
	Socioeconomic status (zip code, insurance)
Pregnancy and Medical History	
	Gravidity and parity
	Estimated due date (based on LNMP or first-trimester USS)
	Height
	Weight
	Past medical history (e.g., diabetes, hypertension, thyroid disease, anxiety/depression, infections)
	Medications during pregnancy and study period (including over-the-counter/vitamins)
	Pregnancy complications (pre-eclampsia, gestational diabetes mellitus, preterm labor, placenta previa, chorioamnionitis)
	Date of hypertension diagnosis
	Hypertensive complications
	Number of hospitalizations for management of hypertension in pregnancy
	Anti-hypertensive medication use and regimens (dose, frequency, dates/times)
	Delivery hospital
Outcomes Data	
	Type of delivery (vaginal, operative instrumental, operative cesarean section)
	Gestational age at delivery
	Race/ethnicity and sex of baby
	Neonatal status (NICU admission; Apgar scores; weight and height at delivery; complications, including SGA, RDS, sepsis, PDA)
